# Lifestyle behaviours associated with 5-year weight gain in a prospective cohort of Australian adults aged 26-36 years at baseline

**DOI:** 10.1186/s12889-016-3931-y

**Published:** 2017-01-10

**Authors:** Kylie J. Smith, Seana L. Gall, Sarah A. McNaughton, Verity J. Cleland, Petr Otahal, Terence Dwyer, Alison J. Venn

**Affiliations:** 1 Menzies Institute for Medical Research, University of Tasmania, Private Bag 23, Hobart, Tasmania 7000 Australia; 2 Institute for Physical Activity and Nutrition, School of Exercise and Nutrition Sciences, Deakin University, Burwood, Victoria 3125 Australia; 3 The George Institute for Global Health, University of Oxford, Oxford, UK

**Keywords:** Skipping breakfast, Takeaway food, Fast food, Television, Steps, Sedentary behaviour, Physical activity, Guidelines, Weight gain, Young adults

## Abstract

**Background:**

Whether not meeting common guidelines for lifestyle behaviours is associated with weight gain is uncertain. This study examined whether 5-year weight gain was predicted by not meeting guidelines for: breakfast consumption (eating between 6 and 9 am), takeaway food consumption (<2 times/week), television viewing (<2 h/day) and daily steps (≥10,000 steps/day).

**Methods:**

One thousand one hundred and fifty-five Australian participants (43% men, 26–36 years) completed questionnaires and wore a pedometer at baseline (2004-06) and follow-up (2009-11). Weight was measured or self-reported, with a correction factor applied. For each behaviour, participants were classified according to whether they met the guideline: consistently met at baseline and follow-up (reference group); not met at baseline but met at follow-up; met at baseline but not met at follow-up; consistently not met at baseline and follow-up. For each behaviour, weight gain was calculated using linear regression. Weight gain by number of guidelines met was also examined.

**Results:**

Mean 5-year weight gain was 2.0 kg (SD:6.3). Compared to the reference group, additional weight (mean, 95% CI) was gained among those who did not meet the guideline at follow-up, or consistently did not meet the guideline, for breakfast (1.8 kg, 0.7–2.9; 1.5 kg, 0.1–2.8); takeaway food (2.2 kg, 0.7–3.6; 1.9 kg, 0.7–3.1); watching television (1.9 kg, 0.9–2.9; 1.4 kg, 0.4–2.3); and daily steps (2.6 kg, 1.1–4.04; 1.6 kg, 0.5–2.7). Those who met ≤1 guideline at follow-up gained 3.8 kg (95% CI 2.3–5.3) more than those meeting all guidelines.

**Conclusion:**

Individuals who adopted healthier behaviours between baseline and follow-up had similar weight gain to those who met the guidelines at both time points. Encouraging young adults to meet these simple guidelines may reduce weight gain.

**Electronic supplementary material:**

The online version of this article (doi:10.1186/s12889-016-3931-y) contains supplementary material, which is available to authorized users.

## Background

Obesity is associated with an increased risk of developing cardiovascular disease, type 2 diabetes, arthritis and some cancers [1]. However, weight gain is common during young adulthood [2, 3]. Weight gain may be due to genetic and behavioural factors such as high-energy intake and low levels of physical activity [1]. To promote healthy eating and increase physical activity, the behaviours typically associated with healthy body weight, key agencies have issued guidelines on common behaviours that can be disseminated in simple and easily understood public health messages. Examples of behaviours that are promoted include ‘eat breakfast’ [4], ‘limit takeaway and fast food to once per week’ [5], ‘watch television less than 2 h per day’ [6] and ‘take at least 10,000 steps per day’. Whether achieving simple guidelines helps to prevent weight gain or if weight gain is greater among those who consistently do not meet guidelines, has not been well examined.

Low health literacy is common, with an estimated 90 million adults from the USA thought to have difficulty understanding and following health information [7]. Basing recommendations on a simple behaviour that individuals recognise may be more effective than focusing on the more complex behaviour. For example providing people with a pedometer and a daily steps goal (which has been shown to increase physical activity) [8], may make it easier for people to monitor their physical activity than more complex guidelines concerning duration, frequency and intensity of physical activity [9].

The 2010 Dietary Guidelines for Americans recommend eating breakfast [4]. Skipping breakfast has been reported to be associated with overweight and obesity [10]. Few longitudinal studies have examined the association between breakfast skipping and weight gain among adults and the results have been inconsistent, with studies reporting skippers have higher [11, 12], lower [13] or similar [14] weight gain to those who eat breakfast. Change in breakfast habits may be important. Two randomised trials reported that individuals who were randomised to the breakfast intervention that was different to their normal breakfast habits had lower energy intakes [15] and lost more weight [16] than those who did not change their breakfast habits.

The National Heart Foundation of Australia recommends limiting takeaway and fast food to no more than once per week [5]. A review article examining the health effects of takeaway and fast foods reported that consumption of these foods was associated with a higher risk of being overweight or obese [17]. In cross-sectional studies, participants who consumed takeaway food or fast food at least twice per week had a higher likelihood of having abdominal obesity [18] and being obese [19] than those who ate takeaway and fast food no more than once per week.

Sedentary behaviour and physical activity have been shown to be associated with weight gain, independently of each other [20]. Watching less than two hours of television each day is recommended by the National Heart Foundation of Australia [6]. Television viewing is believed to contribute to weight gain by either displacing physical activity [21] or by the food and beverages consumed while watching television [22]. In cross-sectional analysis, young adults who, on average, watched more than two hours per day of television had a higher likelihood of moderate abdominal obesity than those who watched less than two hours [22].

Pedometers provide an objective measure of physical activity, are relatively cheap and easy for the general public to use. Although there is yet to be international consensus on the most appropriate number of daily steps for health benefits, 10,000 steps is commonly recommended [23, 24]. The guideline of 10,000 steps/day first originated in Japan but was not evidence based at the time [25]. A five-level index with 10,000 steps indicating the transition to an ‘active’ state, has proven useful for classifying cardiometabolic risk [20]. At the end of a 4-month pedometer workplace challenge, those who averaged at least 10,000 steps/day had a greater reduction in waist circumference, compared with those who did not meet the guideline [26].

The aim of this study was to examine whether meeting simple guidelines of ‘eat breakfast’ [4], ‘limit takeaway and fast food to once per week’ [5], ‘watch television less than 2 h per day’ [6] and ‘take at least 10,000 steps per day’, was associated with 5-year weight gain among young adults. This study builds on previous research by examining four behaviour guidelines in the same sample. Because behaviour can change over time, we examined whether not meeting the guidelines at baseline and/or follow-up resulted in greater weight gain.

## Methods

**Fig. 1:**
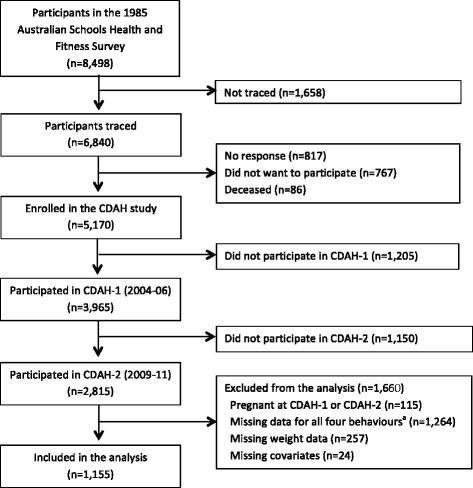
Inclusion and exclusion of participants from the Childhood Determinants of Adult Health (CDAH) study. ^a^Participants who were originally non-responders were given the option of doing a shorter version of the questionnaires, which did not include the breakfast, takeaway food or television questions and they were not asked to wear a pedometer. Participants were included in the analysis if they had baseline and follow-up data for at least one of the behaviours of interest

The data come from the Childhood Determinants of Adult Health (CDAH) study, which is a follow-up of the 8,498 children who participated in the 1985 Australian Schools Health and Fitness Survey (ASHFS) [27]. ASHFS was a nationally representative sample of 7–15 year old Australian children. During 2002-04, 6,840 participants were successfully traced and 5,170 were enrolled in the CDAH study (Fig. 1). During 2004-06, data for the first follow-up (CDAH-1) were collected when participants were 26–36 years old. 2,410 participants attended one of 34 study clinics held in each state and territory of Australia, which included physical measurements and completion of questionnaires. An additional 437 participants completed the questionnaires but did not attend a clinic. For these analyses, the CDAH-1 measures are considered baseline data. During 2009-11, the second wave of follow-up (CDAH-2) was conducted, when participants were 31–41 years old. This follow-up consisted of postal questionnaires. The study protocols were approved by the Southern Tasmanian Health and Medical Ethics Committee and written informed consent was obtained by all participants at both time points.

### Skipping breakfast

Skipping breakfast was assessed at baseline and follow-up using a meal patterns chart, which divided the day into hourly intervals from 6 am–11 pm with the hours from 11 pm–6 am combined into one time interval. Participants were asked to recall the previous day, and for each time interval they were asked ‘Did you eat anything?’ The response options were ‘no’, ‘a snack’, ‘a small meal’, or a ‘large meal’. Participants were also asked ‘Did you drink anything?’ with response options ‘no’, ‘water’, ‘alcohol’ or ‘something else’. The day of the week that the chart was referring to was recorded and dichotomised to weekday or weekend day. Skipping breakfast was defined as not eating a snack, small meal or large meal between 6 and 9 am [28]. The 2010 Dietary Guidelines for Americans recommend eating breakfast [4].

### Takeaway food consumption

Takeaway food consumption was estimated at both time points using the question ‘How many times per week would you usually eat hot takeaway meals? (e.g. pizza, burgers, fried or roast chicken, Chinese/Indian/Thai takeaway)’. Response options ranged from ‘I don’t eat takeaway’ to ‘6–7 meals per week’. This question has been shown to be a valid measure of takeaway food consumption [18]. Takeaway food consumption was dichotomised and those who consumed takeaway once per week or less were classified as meeting the guideline, as recommended by the National Heart Foundation of Australia [5].

### Television viewing

Participants were asked to estimate the total time during the last week that they spent watching television, videos, or DVDs when it was the main activity. Total time (hours and minutes) was reported separately for weekdays and weekend days [29]. The times for weekdays and weekend days were summed, converted to hours per week and then divided by seven to give the average daily television viewing time. Daily television viewing was dichotomised and participants who watched less than two hours/day were classified as meeting the guideline, as recommended by the National Heart Foundation of Australia [6]. Television viewing was chosen as a measure of sedentary behaviour, rather than total sitting time, because television viewing is a modifiable leisure-time behaviour (and hence amenable to intervention), whereas the amount of time spent sitting can be strongly influenced by occupation.

### Daily steps

Participants were asked to wear a pedometer (Yamax Digiwalker SW200) for seven days and to complete a pedometer diary. At baseline, a clinic staff member demonstrated the correct placement of the pedometer and how to reset it. Written instructions were provided at both time points. At the end of each day participants recorded the number of steps taken, the time that they put the pedometer on and when they took it off. The pedometer and diary were returned via post. When the pedometer was returned, the battery was checked and the pedometer was calibrated. Yamax pedometers have been reported to be one of the most accurate brands of pedometer [30]. The average number of daily steps were calculated for participants who reported wearing the pedometer for at least 8 h on at least four days. Average daily steps were dichotomised and participants who did ≥10,000/day were classified as meeting the guideline. There is some debate around the number of steps required each day, with 10,000 steps being the most widely used [23].

### Body weight

At the CDAH-1 study clinics, weight and height were measured using a portable digital scale (Heine, Dover, NH) and a portable stadiometer (Invicta, Leicester, UK). The scales were calibrated by an external calibration service four times during the study. Participants wore light clothing and no shoes. Weight and height were self-reported by those who did not attend a study clinic at CDAH-1 (*n* = 11) and by all participants at CDAH-2. BMI (kg/m^2^) was calculated. A subset of 1,185 participants had both self-reported and measured weight and height at the CDAH-1 clinic and a correction factor was derived using linear regression. The correction factor was applied to weight and BMI values calculated from self-reported data at CDAH-1 and CDAH-2 [31]. The correlation between measured and self-reported weight at baseline was 0.95. Change in weight was calculated as weight at follow-up minus weight at baseline.

### Covariates

At baseline participants reported their age and highest level of education (school only, vocational, university) and marital status. At CDAH-2, participants were asked to report the month and year of birth for all biological children. Whether the participant had children at baseline (parental status) was calculated using the child’s date of birth and the date the participant completed the questionnaire at CDAH-1. Parental status was considered as a covariate because having children has been shown to be associated with both healthy [32] and unhealthy dietary [33] changes and reduced physical activity levels [33, 34], which may lead to a change in weight. Time to follow-up was calculated as the number of days between completing the baseline and follow-up questionnaires. Leisure time physical activity and sitting time were estimated using the long version of the International Physical Activity Questionnaire (IPAQ) [35]. Smoking status was self-reported at CDAH-1 and CDAH-2 and a change in smoking status variable was created (non-smoker, started smoking, quit smoking and persistent smoker). Participants completed a 127-item food frequency questionnaire that estimated dietary intake over the previous year and a food habits questionnaire [18, 36]. Alcohol intake (g/week) was calculated from nine alcoholic beverages in the food frequency questionnaire and their average alcohol content [37]. Diet quality at baseline was estimated using a 15-item dietary guideline index, based on the Dietary Guidelines for Australian Adults [38] and the Australian Guide to Healthy Eating [39]. For each age and sex-specific dietary guideline, participants were awarded 0–10 points, with ten indicating greatest compliance (possible score range 0–150) [40].

### Statistical analysis

Chi-square tests and t-tests were used to compare baseline characteristics of those who were included in the analyses to those who were excluded or did not participate at follow-up. For each lifestyle behaviour, participants were classified into one of four groups according to whether or not they met the guideline: consistently met at baseline and follow-up (reference group), not met at baseline but met at follow-up (adopted healthier behaviour), met at baseline but not at follow-up (developed unhealthy behaviour), consistently not met at baseline and follow-up. Linear regression models with the outcome of follow-up weight adjusted for baseline weight were used to examine if 5-year weight change differed among participants who did not meet the recommendations compared to those who consistently met the guidelines. Each behaviour was examined separately. Model 1 adjusted for sex, baseline weight and time to follow-up. Model 2 adjusted a priori for sex, baseline weight, time to follow-up, and baseline age. When skipping breakfast was the exposure of interest, model 2 was further adjusted for day of the week that the meal pattern chart was completed (week day/weekend) because participants were more likely to skip breakfast on a weekend day than on a weekday. Other covariates considered for inclusion in model 2 included baseline education, marital status, parental status, leisure time physical activity, alcohol intake, time spent sitting and change in smoking status. These additional covariates were only included in model 2 if they changed the beta coefficient for the exposure of interest by at least 10%. To determine if there were differences in 5-year weight gain between those who were normal weight or overweight/obese at baseline, an interaction term was included in the models.

Two sensitivity analyses were performed. We have previously shown that skipping breakfast [28] and frequent takeaway food consumption [18] are associated with poorer diet quality in this sample. Therefore diet quality (estimated using the dietary guideline index) was added to the model to examine whether it mediated the associations between behaviour and weight gain (model 3, Additional file 1). Loss to follow-up does necessarily result in selection bias [41] but to examine its impact on our results, if any, we weighted participants by the inverse of the probability of not being missing so that each participant represented non-participants similar to them (inverse propensity weighting) [42]. CDAH-1 variables that differed between participants and non-participants were used in the propensity model: sex, age, education, marital status, smoking status and body weight.

All statistical analyses were conducted using STATA software (version 12.0, 2011, Statacorp, College Station, Texas). *P*-value ≤0.05 was considered statistically significant. Men and women were analysed together and sex interactions between behaviour and weight gain were examined.

## Results

In total, 2,815 participants completed both baseline and follow-up measures (Fig. 1). Participants were excluded from the analysis if they were pregnant at baseline or follow-up (*n* = 115), missing data on all four behaviour variables (*n* = 1,264), missing weight data (*n* = 257) or any of the covariates included in the models (*n* = 24). This left 1,155 individuals who had data at both time points for at least one of the behaviours of interest.

Baseline characteristics of the sample are reported in Table 1. Over half the men (57.4%) and one-third of women (35.6%) were overweight or obese at baseline. The percentage meeting each of the guidelines was higher for women than for men, with the exception of daily steps where the percentage was similar for men and women. Of those who were classified as breakfast skippers, 60% reported having a drink between 6 and 9 am at baseline and follow-up. Two participants who skipped breakfast reported drinking alcohol at this time, one at baseline and one at follow-up.

**Table 1:** Baseline sociodemographic, weight status and behaviour characteristics for Australian men and women aged 26–36 years

	Men (*N* = 492)^a^		Women (*N* = 664)^a^	
Characteristic	*n*	%	*n*	%
Age (mean, SD)	31.65	(2.54)	31.31	(2.67)
Education				
University	212	43.1	352	53.0
Vocational	170	34.6	151	22.7
School only	110	22.4	161	24.3
Marital status				
Single	159	32.3	188	28.3
Married or living as married	333	67.7	476	71.7
Parental status				
No children	303	62.1	335	50.5
Children	185	37.9	329	49.6
Occupation				
Professional or manager	295	61.6	345	52.9
Non-manual	37	7.7	169	25.9
Manual	130	27.1	22	3.4
Not in the workforce	17	3.6	116	17.8
Weight status				
Normal (<25 kg/m^2^)	210	42.7	428	64.5
Overweight (≥25–<30 kg/m^2^)	212	43.1	156	23.5
Obese (≥30 kg/m^2^)	70	14.2	80	12.1
Ate breakfast				
Yes	337	70.5	515	79.2
No	141	29.5	135	20.8
Ate takeaway <2 times/week				
Yes	311	64.5	542	83.0
No	171	35.5	111	17.0
Television viewing <2 h/day				
Yes	245	51.4	433	66.1
No	232	48.6	222	33.9
≥10,000 steps/day				
Yes	155	34.6	198	32.8
No	293	65.4	405	67.2

^a^Due to missing data, numbers do not always equal the total

**Fig. 2:**
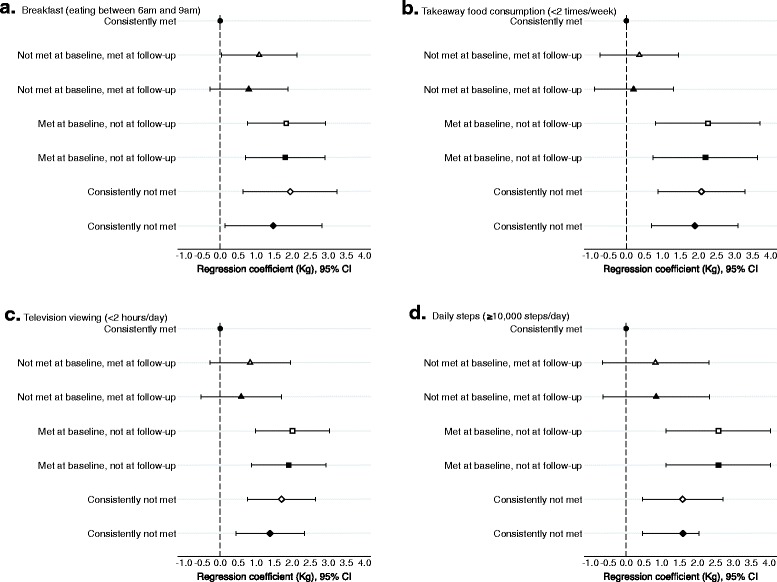
Mean difference in 5-year weight change (kg) among Australian adults aged 26–36 years at baseline, by whether they met the behaviour guidelines at baseline and follow-up. **a** Breakfast (eating between 6am and 9am); **b** Takeaway food consumption (<2 times/week); **c** Television viewing (<2 hours/day); **d** Daily steps (≥10,000 steps/day). This figure illustrates the differences in weight change for those who did not met the guidelines (met at follow-up but not at baseline, met at baseline but not at follow-up, consistently not met) compared with those who met the guidelines at both time points (reference group). Results to the right of the y-axis indicate greater weight gain compared to the reference group and to the left indicate less weight gain. If the error bars cross the y-axis the results are not significantly different to the reference group. Model 1 (*open symbol*): Adjusted for sex, baseline weight and time to follow-up. Model 2 (*closed symbol*): *Breakfast* - model 1 + age, education, change in smoking status, day that the meal patterns chart was completed at baseline and follow-up (weekend or weekday); *Takeaway food* - model 1 + age, parent status, and change in smoking status; *Television viewing* - model 1 + age, education, parent status, change in smoking status; *Steps* - model 1 + age. Sample sizes: *Breakfast* Consistently met guidelines *n* = 656; Not met at baseline met at follow-up *n* = 168; Met at baseline not at follow-up *n* = 155; Consistently not met *n* = 100. *Takeaway food* Consistently met guidelines *n* = 761; Not met at baseline met at follow-up *n* = 151; Met at baseline not at follow-up *n* = 79; Consistently not met *n* = 124. *Television viewing* Consistently met guidelines *n* = 435; Not met at baseline met at follow-up *n* = 165; Met at baseline not at follow-up *n* = 204; Consistently not met *n* = 277. *Steps* Consistently met guidelines *n* = 148; Not met at baseline met at follow-up *n* = 114; Met at baseline not at follow-up *n* = 121; Consistently not met *n* = 465

Mean follow-up time was 4.98 (SD 0.31) years. The number of participants in the four guideline categories for each behaviour are reported in Fig. 2. The majority of participants consistently met the recommendations for breakfast, takeaway food consumption and television viewing. In contrast, the majority of participants consistently did not meet the guideline for ≥10,000 steps/day. For skipping breakfast and daily steps, the number of participants who adopted the healthier behaviour (guideline not met at baseline but met at follow-up) and developed the unhealthy behaviour (met the guideline at baseline but not at follow-up) was similar. For takeaway food consumption, the number of participants who adopted the healthier behaviour was higher than the number who developed the unhealthy behaviour whereas for television viewing more participants developed the unhealthy behaviour than adopted the healthier behaviour.

During the 5-year follow-up, the mean (SD) weight gain was 2.0 kg (6.2) for men and 2.0 kg (6.4) for women. One hundred and thirty (26.4%) men and 166 (25%) women gained at least 5 kg. Weight loss also occurred, with 52 (10.6%) men and 62 (9.3%) women losing at least 5 kg.

Compared to those who met the guidelines at both time points, there was significantly greater weight gain among individuals who did not meet each of the recommendations at follow-up (developed unhealthy behaviour, or consistently not met) after adjusting for covariates (Fig. 2). The greatest weight gain tended to be among those who developed the unhealthy behaviour (met the guideline at baseline but not at follow-up). Individuals who adopted the healthier behaviour (did not meet the guideline at baseline but met at follow-up) did not differ significantly in weight gain from those who consistently met the guideline. There were no interactions between behaviour and weight gain for sex or baseline weight status.

**Fig. 3:**
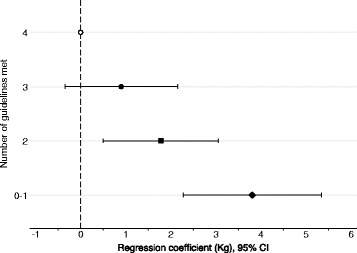
Mean difference in 5-year weight change (kg) among Australian adults aged 26–36 years at baseline, by the number of behaviour guidelines that participants met at follow-up. Analysis adjusted for sex, baseline weight, age and time between baseline and follow-up. Sample size: met 4 behaviours *n* = 135; 3 behaviours *n* = 360; 2 behaviours *n* = 323; 0–1 behaviours *n* = 136

A post-hoc analysis was conducted to examine if there was a cumulative effect, with greater weight gain among those who were not meeting multiple guidelines. Behaviour at follow-up explained most of the weight gain therefore only the four behaviours at follow-up were used. We combined those who met none or only one guideline, as only 18 participants did not meet any of the guidelines. Weight gain was negatively associated with the number of guidelines met (Fig. 3). Compared to those who met all four guidelines, those meeting none or only one guideline gained an extra 3.8 kg (95% CI 2.3, 5.4) while individuals who only met two guidelines gained an extra 1.8 kg (95% CI 0.5, 3.1). Weight gain among those who met three guidelines (0.9 kg 95% CI -0.3, 2.2) was not significantly different to those who meet all four guidelines.

We also compared the number of guidelines met among those who lost weight. There were 173 participants who lost weight (at least 2 kg) from baseline to follow-up and had data for all four behaviours. Those who met more of the guidelines tended to lose more weight than those who met only one or none of the guidelines but the differences were not statistically significant, possibly due to the small sample size. Compared with those who met none or only one guideline (*n* = 19), more weight was lost by those who met all four guidelines (*n* = 24) (mean:-0.5 kg; 95% CI -1.9, 0.9) and those who met three guidelines (*n* = 69) (mean: −0.5 kg; 95%CI −1.6, 0.7). Those who met two guidelines (*n* = 61 had similar weight loss to those who meet none or only one guideline (mean: 0.2 kg; 95% CI −0.9, 1.3).

There were some differences between participants who were included in the analysis and non-responders (did not participate in the CDAH-2 follow-up or were missing data). Compared to those who participated in the follow-up (see Table 1), non-responders (data presented in parentheses) were less likely to be university educated (29.1% men, 36.3% women), men who did not participate were more likely to be single (35.6%), and women were more likely to skip breakfast (27.4%). Responders and non-responders were similar regarding the percentage of women who were single (28%), men who skipped breakfast (32.5%), the percentage who ate takeaway ≥2 times/week (39.3% men, 18.2% women), watched ≥2 h/day of television (47.4% men, 38.4% women) and averaged <10,000 steps/day (60.8% men, 70.6% women). Mean BMI was similar between those included in the analysis (26.1 kg/m^2^ (SD 4.2) for men, 24.7 kg/m^2^ (SD 4.9) for women) and non-responders (26.6 kg/m^2^ (SD 4.3) for men, 25.2 kg/m^2^ (SD 5.6) for women).

The baseline characteristics of the participants were also compared to the general population of Australian adults of a similar age. Compared to the general population, a higher percentage of the CDAH sample were married or living as married (57% men, 64% women in the general population [43]) or employed as professionals or managers (40% men, 38% women [44]). The percentage classified as being overweight or obese was very similar to the general population (58% men, 35% women [45]).

### Sensitivity analyses

Diet quality did not appear to explain the association between behaviour and weight gain. Only the association between takeaway food consumption and weight was slightly attenuated when diet quality was included in the model (Additional file 1). When the data were weighted to take into account loss to follow-up, the size of the effect was attenuated for those who skipped breakfast at follow-up (from 1.8 to 1.1 kg) or both time points (from 1.5 to 1.1 kg), and ate takeaway ≥2 times/week at follow-up (from 2.2 to 1.9 kg, Additional file 2). The strength of the association increased for those who ate takeaway ≥2 times/week at both time points (from 1.9 to 2.2 kg, Additional file 2). For the other associations, the effect sizes for the weighted data were similar to the unweighted data (Additional file 2).

## Discussion

This study aimed to examine whether not meeting simple guidelines for eating breakfast, takeaway food consumption, television viewing and daily steps at baseline and/or follow-up predicted greater 5-year weight gain. Behaviour at follow-up was found to be the most important in terms of weight gain, with participants who did not meet the guideline at follow-up (consistently not met, and developed unhealthy behaviour) gaining significantly more weight than those who consistently met the guideline. Participants who developed unhealthy behaviours during the 5-year period (met the guideline at baseline but not at follow-up) tended to have the greatest weight gain. Weight gain increased as the number of guidelines met decreased. Participants who adopted healthier behaviours between baseline and follow-up (did not meet the guideline at baseline but met at follow-up) had similar weight gain to those who consistently met the guidelines.

During the 5-year follow-up, the mean weight gain was 2.0 kg for both men and women. This is slightly lower than observed in other studies with participants of similar age and follow-up duration. In the Australian Diabetes, Obesity and Lifestyle (AusDiab) study, a national sample of Australian adults, participants aged 25–34 years at baseline (1999–2000) gained an average of 3.4 kg for men and 3.5 kg for women during the 5-year follow-up [46]. In the Young Finns study, among those who were 24–39 years old at baseline (2001), mean 6-year weight gain was 2.7 kg for women and 3.5 kg for men [47].

Consistently skipping breakfast and becoming a breakfast skipper were associated with greater weight gain in this study. Previous studies have reported that individuals who skip breakfast tend to have poorer diet quality [28, 48] and higher energy intakes [49] than those who eat breakfast. Diet quality did not explain the greater weight gain observed in this study and we were unable to adjust for energy intake. The proportion of daily energy intake consumed at breakfast may be important. A study of middle aged adults found those who consumed a higher percentage of their daily energy intake at breakfast had lower weight gain during the mean follow-up period of 3.7 years [50]. Types of foods consumed at breakfast may also be important, with individuals who consume ready-to-eat cereals for breakfast being less likely to be overweight or obese than to those who eat other breakfast foods [51].

Skipping breakfast may also be a marker of other lifestyle factors that are linked to weight gain, such as lower levels of physical activity [28, 52]. In addition, there may be a physiological effect, where prolonging the overnight fast may affect metabolism; however, no metabolic mechanism has yet been identified.

Frequent takeaway food consumption was also associated with weight gain, which is consistent with previous studies reporting takeaway and fast foods are associated with a higher risk of being overweight or obese [17]. We have previously shown that frequent takeaway food consumption is associated with a poorer diet quality in this sample [18]. Adjusting for diet quality slightly reduced the magnitude of the effect, particularly for those who consistently ate takeaway food ≥2 times/week. Takeaway food items are often energy dense [53] and energy intake has been reported to be higher on days when fast food is consumed than non-fast food days [54]. The amount of energy consumed per eating occasion tends to be higher for foods purchased away from the home than foods prepared at home [55]. Energy labelling of takeaway and fast food may help raise awareness of the high-energy content of these items. One systematic review found that calorie labelling alone did not reduce the number of calories customers selected or consumed. However, additional information, such as reporting the recommended daily calorie intake or a symbol for low fat options, was associated with selecting and consuming lower energy meals [56]. Greater promotion of healthier takeaway options may be needed to encourage customers to make healthier choices, as an Australian study reported that healthier options were not very popular, with only 1% of meals (11 out of 1448) purchased from the ‘healthy’ menu [57].

Legislation requiring takeaway and fast food outlets to report the energy content of products may encourage the industry to provide lower energy options and there is some evidence that this is already occurring [58]. Reducing portion sizes for takeaway and fast food may be important as there is evidence that larger amounts of food are consumed when greater amounts are served [59]. Reducing serving sizes and changing the composition of meals to include lower calorie options could significantly decrease the energy content of takeaway food purchases [60]. There is wide variation in the nutrition content for similar products across companies, suggesting that changes could be made to a large number of items to improve their nutritional value [61].

Watching television for two or more hours per day, particularly at follow-up, was also associated with greater weight gain, as supported by other studies [62]. Television viewing may be associated with weight gain through unhealthy consumption of snacks while watching television and increased exposure to advertising of unhealthy products. Food advertising can prime automatic eating behaviours and promote eating of products other than those being advertised [63]. Television advertisements have also been reported to increase desire to eat among overweight and obese individuals, but not healthy weight individuals [64]. Distracted eating is also a possibility, where individuals over-consume because they are not paying attention to what they are eating.

Television viewing may also displace physical activity, however, leisure-time physical activity was not found to be a confounder in our study. Sitting behaviours tend to displace light intensity physical activity rather than moderate-to-vigorous physical activity [65], so sitting behaviour may not have a large impact on weight. Very few studies that have examined the association between television viewing and weight gain have measured both eating behaviours while watching television and physical activity, so it is not clear whether weight gain is better explained by eating behaviours or reduced physical activity. In cross-sectional analyses of the CDAH data, the association between television viewing and abdominal obesity was not explained by leisure time physical activity and only partially explained by food and beverages consumed while watching television, suggesting there may be other mechanisms present [22].

Of the four behaviours we examined, taking ≥10,000 steps/day was the guideline that was least likely to be met and the greatest weight gain was observed among those who became non-compliant with the guideline. These findings are important because walking is a relatively easy, cheap and accessible activity to promote and is the most common physical activity undertaken among adults in Australia [66] and other countries [67]. In addition, walking can be incidental and accumulated throughout the day. The Australian Physical Activity Guidelines recommend simple ways to increase physical activity in daily tasks such as taking the stairs instead of the elevator, parking further away from the destination, or getting off public transport one or two stops earlier, and walking the rest of the way [9]. In order for individuals to undertake these activities, it is important to create physical and policy environments that support individuals to be physically active in their everyday tasks and further research is needed to identify ways to increase the uptake of these messages.

There are several limitations of this study that should be considered when interpreting these findings. Data were collected at only two time points, 5 years apart, and it is not known when the change in behaviour occurred. If participants changed their behaviour just before the second follow-up then this might underestimate the effect as the behaviour had less time to impact on weight. Weight was self-reported by some participants at baseline and all participants at follow-up, however, the correlation between measured and self-reported weight at baseline was excellent and we were able to adjust the self-reported data using a correction factor calculated from a subsample of participants who had both self-reported and measured weight at baseline. The FFQ did not include options for serving sizes therefore a measurement of energy intake was not available. Although having a measure of energy intake would be beneficial to examine if eating behaviours were associated with higher energy intakes, estimates of energy intake can introduce more error, as participants are required to estimate not only how often they consume each item but also how much, on average, they consume. This amount is then multiplied by an average amount of energy from a variety of similar products. The study sample was of higher socioeconomic status than the general Australian population, which may limit the generalisability of the findings. While a nationally representative sample is very important in a prevalence study, a lack of representativeness should not affect the associations observed between behaviour and weight change provided that information on the relevant confounders is well distributed and adjusted for in the analysis.

Skipping breakfast was defined using data from the previous day, which might not be typical of the participant’s normal breakfast eating habits. Participants who ate breakfast before 6 am would have been misclassified as breakfast skippers if they did not eat again before 9 am. Late risers and shift workers may also have been misclassified however, the majority (60%) of those classified as skipping breakfast reported having something to drink between 6 and 9 am indicating they were awake at that time. In addition, the Dietary Guidelines for Americans recommend eating breakfast but do not specifically state to eat it everyday. There are also other eating behaviours not included in this study, such as consumption of fruit and vegetables, alcohol, and sugar-sweetened beverages, which may impact on weight gain. Pedometers are unable to measure physical activity that does not generate steps, such as swimming, cycling or upper body workouts. Therefore individuals who were active in non-step taking activities may have been classified as taking <10,000 steps/day, resulting in underestimation of the effect on weight of not meeting the guideline for daily steps. In addition, television viewing was our measure of sedentary behaviour, which did not include other screen-based activities such as computers, tablets or smartphones, which have become increasingly popular and therefore the effect of sedentary behaviour on weight gain may be underestimated. There was a large loss to follow-up and accounting for loss to follow-up using inverse propensity weighting attenuated some of the results. Due to the observational study design there is the potential for unmeasured confounding to introduce error in our estimates.

The strengths of this study include the longitudinal data, examining change in behaviour and the ability to examine a range of different weight-related behaviours in the same sample. Using pedometers provides an objective measure of physical activity, as pedometers pick up short periods of activity or incidental activity that may not be reported in questionnaires. Sedentary behaviours, such as television viewing, are receiving great attention in the literature at present, but most work to date is cross-sectional and remains in its infancy [68].

## Conclusion

Meeting simple dietary, sedentary behaviour and physical activity guidelines was associated with less weight gain over 5 years in these young adults. Among the 13–16% of individuals who adopted healthier behaviours, weight gain was similar to those who met the guidelines at both time points, indicating the benefits of behaviour change in this age group. Further research to investigate the individual, social and environmental factors that promote or hinder compliance with public health recommendations for diet, sedentary behaviours and physical activity, is needed to help identify the mechanisms through which these behaviours change over time and identify key areas for interventions. Studies with multiple assessments of behaviour over time are also needed to better understand the timing of changes in behaviour in relation to weight gain.

## Additional files

Additional file 1: Mean difference in 5-year weight change (kg) among Australian adults, by compliance with behaviour guidelines, adjusted for diet quality. (PDF 123 kb)
Additional file 2: Mean difference in 5-year weight change (kg) among Australian adults aged 26–36 years at baseline, by behaviour, adjusting for loss to follow-up using inverse propensity weighting. (PDF 133 kb)

## Abbreviations

ASHFS: Australian Schools Health and Fitness Survey
CDAH: Childhood Determinants of Adult Health
