# Supplementary material for: Lifestyle behaviours associated with 5-year weight gain in a prospective cohort of Australian adults aged 26-36 years at baseline
Source: BMC Public Health. 2017 Jan 10;17:54. doi: 10.1186/s12889-016-3931-y (PMC5223543; doi:10.1186/s12889-016-3931-y)
Supplement: Additional file 1: — Mean difference in 5-year weight change (kg) among Australian adults, by compliance with behaviour guidelines, adjusted for diet quality. (PDF 123 kb) [file 12889_2016_3931_MOESM1_ESM.pdf]

Additional File 1: Mean difference in 5-year weight change (kg) among Australian adults, by compliance with behaviour guidelines, adjusted for diet quality

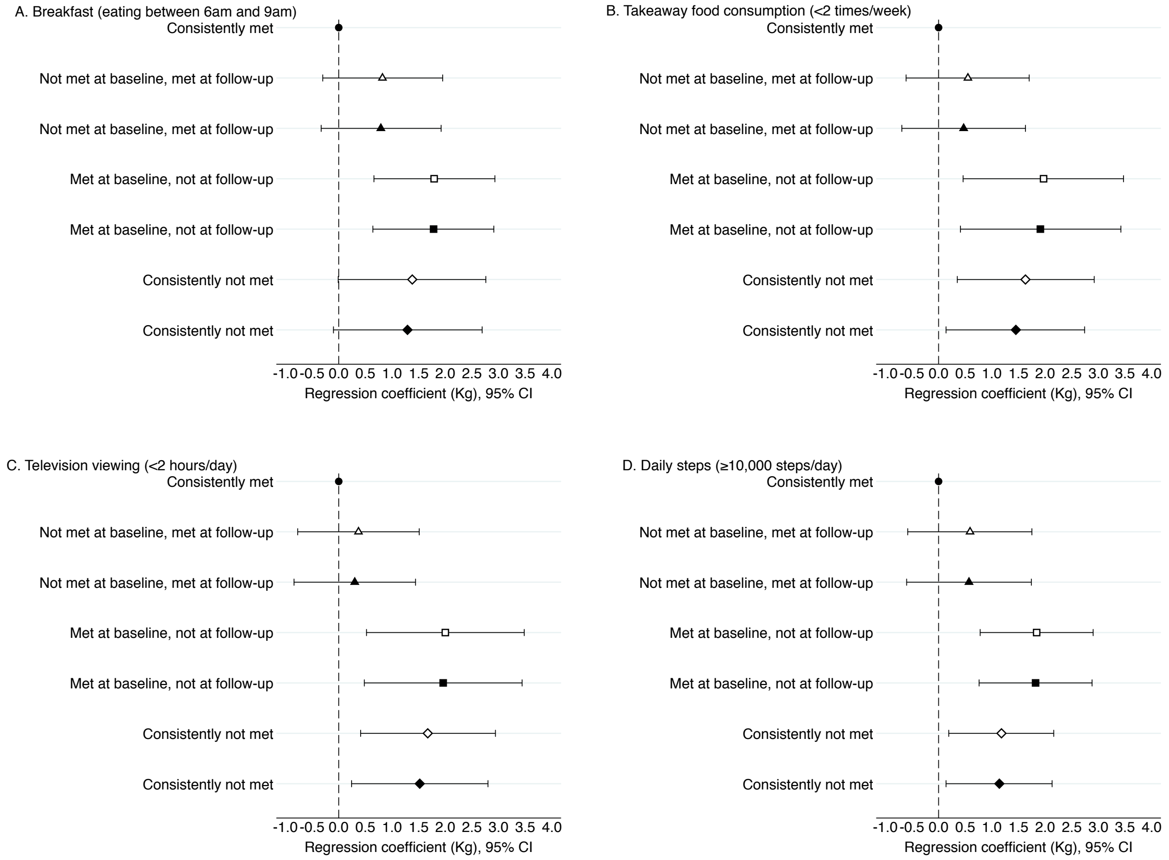

This figure illustrates the difference in weight change for those who did not met the guideline (not met at baseline but met at follow-up, met at baseline not at follow-up, consistently not met) compared to those who met the guideline at both time points (reference group). Results to the right of the y-axis indicate greater weight gain compared to the reference group and to the left indicate less weight gain. If the error bars cross the y-axis the results are not significantly different to the reference group.

Model 2 (open symbol): *Breakfast* - adjusted for sex and baseline weight, age, time to follow-up, education, change in smoking status, day that the meal patterns chart was completed at baseline and follow-up (weekend or weekday); *takeaway food* - adjusted for sex and baseline weight, age, time to follow-up, parent status, and change in smoking status; *television viewing* - adjusted for sex and baseline weight, age, time to follow-up, education, parent status, change in smoking status; *Steps* - adjusted for sex and baseline weight, age, time to follow-up.

Model 3 (closed symbol): Model 1 + additional adjustment for diet quality.
