# Supplementary material for: Lifestyle behaviours associated with 5-year weight gain in a prospective cohort of Australian adults aged 26-36 years at baseline
Source: BMC Public Health. 2017 Jan 10;17:54. doi: 10.1186/s12889-016-3931-y (PMC5223543; doi:10.1186/s12889-016-3931-y)
Supplement: Additional file 2: — Mean difference in 5-year weight change (kg) among Australian adults aged 26–36 years at baseline, by behaviour, adjusting for loss to follow-up using inverse propensity weighting. (PDF 133 kb) [file 12889_2016_3931_MOESM2_ESM.pdf]

Additional File 2: Mean difference in 5-year weight change (kg) among Australian adults aged 26-36 years at baseline, by behaviour, adjusting for loss to follow-up using inverse propensity weighting.

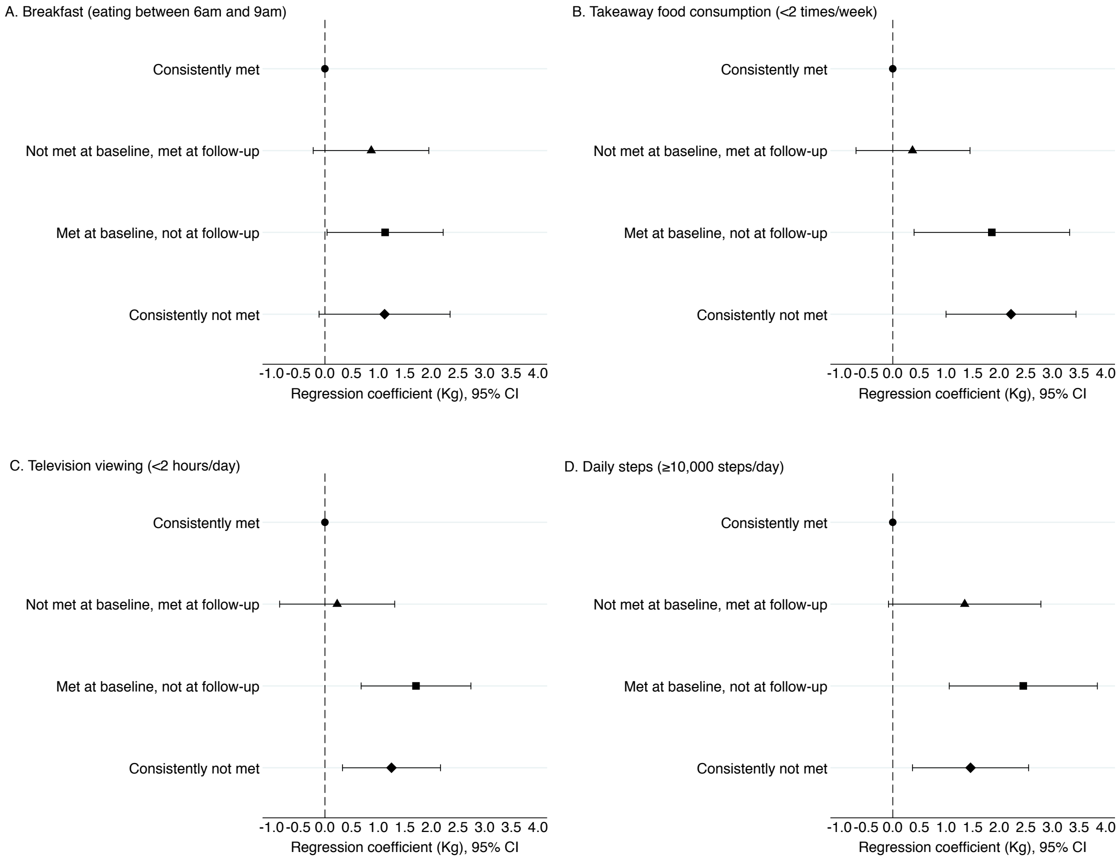

This figure illustrates the difference in weight change for those who did not met the guideline (not met at baseline but met at follow-up, met at baseline not at follow-up, consistently not met) compared to those who met the guideline at both time points (reference group). Results to the right of the y-axis indicate greater weight gain compared to the reference group and to the left indicate less weight gain. If the error bars cross the y-axis the results are not significantly different to the reference group.

Breakfast - adjusted for sex and baseline weight, age, time to follow-up, education, change in smoking status, day that the meal patterns chart was completed at baseline and follow-up (weekend or weekday); *takeaway food* - adjusted for sex and baseline weight, age, time to follow-up, parent status, and change in smoking status; *television viewing* - adjusted for sex and baseline weight, age, time to follow-up, education, parent status, change in smoking status; *Steps* - adjusted for sex and baseline weight, age, time to follow-up.

The propensity model included sex, age, education, marital status, smoking status and body weight.
